# Fear of clowns: An investigation into the aetiology of coulrophobia

**DOI:** 10.3389/fpsyg.2023.1109466

**Published:** 2023-02-02

**Authors:** Philip John Tyson, Shakiela K. Davies, Sophie Scorey, William James Greville

**Affiliations:** School of Psychology and Therapeutic Studies, University of South Wales, Treforest, United Kingdom

**Keywords:** clown fear, coulrophobia, phobia acquisition, phobia, aetiology

## Abstract

**Introduction:**

Fear of clowns or coulrophobia is a little understood phenomenon despite studies indicating that it has a high prevalence in the general population. There have been no previous investigations into the aetiology of this fear, although several plausible hypotheses from the wider literature can be generated; the fear stems from media portrayals of scary clowns, from the unusual physical appearance or the unpredictable behaviour displayed, or it derives from an unpleasant personal experience.

**Methods:**

The current study reviews the literature in this area and also pilots a new questionnaire (Origin of Fear of Clowns Questionnaire; OFCQ) to explore the causes of the fear of clowns in a sample of 528 participants who reported such a fear.

**Results:**

Our findings suggest that uncertainty of harmful intent, media influences and unpredictability of behaviour play an important role in the origins of coulrophobia. There are also multiple features of clown appearance which produce a negative experiential state and a sense of a direct threat.

**Discussion:**

We conclude that the origins of clown fear are multi-factorial and primarily relate to aspects of their facial appearance, their behaviour, and how they have been portrayed in the media. Surprisingly, fear derived from personal experience was not one of our main findings. Further research is focused on looking at associations between the level of fear and each aetiological category.

## 1. Introduction

‘Want your boat, Georgie?’ The clown smiled. […] ‘And a balloon? I’ve got red and green and yellow and blue…’

‘Do they float?’

‘Float?’ The clown’s grin widened. ‘Oh yes, indeed they do. They float! And there’s cotton.

candy …’

George reached.

The clown seized his arm.

And George saw the clown’s face change.

What he saw then was terrible enough to make his worst imaginings of the thing in the.

Cellar look like sweet dreams; what he saw destroyed his sanity in one clawing stroke.

[…] ‘They *float*,’ it growled, ‘they *float*, Georgie, and when you are down here with me, you’ll.

float, too—’

*IT* (King, 2016)

In contemporary Western societies, clowns are typically depicted as friendly figures of fun and comedy, perhaps best embodied in those you would find in a circus or at a children’s party. Yet, the seemingly pleasant circus clown can just as easily upset as entertain, for example if coaxing a shy and embarrassed child to be part of a magic trick, or with a surprise jet of water from a lapel flower. More generally, the erratic behaviour displayed by clowns in their slapstick comedy combined with their outlandish appearance can put an audience on edge; perhaps intentionally. Indeed, throughout history, clowns have held a more ambiguous and multifarious role than simple entertainer. Durwin (2004) and Bala (2010) posit that clowns (and similar incarnations such as the fool, jester or joker) embody the “trickster” archetype, a force that seeks to balance good and evil and that can thus be either benevolent or malign seemingly at whim (a classic example being the god Loki in Norse mythology). Uncertainty thus exists as to the potential of a clown to harm as well as charm.

According to Stott (2012), the development of the clown into an overtly malevolent figure began towards the end of the 19th century, perhaps most prominently in the 1892 opera Pagliacci, in which the central character Canio murders his wife and her lover whilst dressed as a clown. From these earlier depictions emerge the modern trope of ‘killer clowns,’ as exemplified in the above quote featuring the clown Pennywise from the Stephen King novel *It* (1986) and subsequent film and TV adaptations. Such sinister clowns now abound in popular culture (prominent examples include the possessed clown doll in the 1982 movie Poltergeist; and Batman’s nemesis, the Joker) and indeed have had real-life counterparts (the serial killer John Wayne Gacy).

Are these murderous manifestations of clowns in popular culture largely responsible for generating fear of clowns in the general population? Or are they merely the embodiment of an extant fear? This paper sought to investigate the putative origins of *coulrophobia* – the fear of clowns.

### 1.1. The prevalence of clown fear

Coulrophobia is not a well-understood phenomenon and is not explicitly identified as a specific phobia in the DSM-V (American Psychiatric Association, 2013), despite studies indicating that it is present amongst both adults and children, and is represented across many different cultures (Loidl-Keil et al., 2004; Glasper et al., 2007; Seim and Spates, 2010; Barkmann et al., 2013; Meiri et al., 2017; van Venrooij and Barnhoorn, 2017; Rapoport and Berta, 2019; Amirazizi, 2021; Tyson et al., 2022). Although the prevalence of coulrophobia is inconsistent across studies, the largest and most recent survey to date, including 987 adult participants from 64 different countries, reported that 53.5% (528) of their sample had some degree of fear of clowns (Tyson et al., 2022). Other prevalence studies with adult populations have reported lower levels of clown fear: 17% (Rapoport and Berta, 2019); 5.6% (Amirazizi, 2021); 1.5% (Seim and Spates, 2010).

Research involving children and young people has exclusively focussed on a fear of medical clowns, who are employed to entertain young hospitalised patients. Here, between 1 and 6% of those surveyed have expressed apprehension to such characters (Glasper et al., 2007; Barkmann et al., 2013; Meiri et al., 2017). Other studies with similar participants, whilst not reporting frequency data, also acknowledge a dislike of clowns in their samples (Loidl-Keil et al., 2004; Curtis et al., 2007). Interestingly, two of the studies which reported a fear of medical clowns in hospitalised children, also found that such a fear was present amongst the parents of the young patients and the medical staff, with prevalence rates of between 18 and 46% (Glasper et al., 2007; van Venrooij and Barnhoorn, 2017).

These considerable fluctuations in prevalence rates reported across different studies are in part attributable to the severity of fear being documented in each case (see Tyson et al., 2022 for further discussion). Regardless of the exact figures, there is clear evidence that coulrophobia affects a significant portion of the populace, and one key unanswered question relates to the origins and causes of this fear.

### 1.2. Aetiological theories of clown fear

Theories of the aetiology of clown fear can be broadly split into three general categories: those relating to their physical appearance, those relating to their behaviour and those derived from learning and/or experience.

Firstly, considering fear of clowns as deriving from aspects of physical appearance, Moore (2012) suggests that coulrophobia may stem from the uncanny valley effect which describes the feelings of eeriness and repulsion triggered by near human-looking objects. This theory was originally postulated by Mori (1970) and Mori et al. (2012), who speculated about the uneasy feelings which might be elicited when someone touches a cold and limp prosthetic hand. It looks real, but does not possess all the qualities expected for a real human hand, such as warmth and visible bone structure. In contrast, a simple mechanical hand or a genuine human hand do not engender such negative feelings. Therefore, the quality of being ‘near-human’ is the key element which causes uneasiness, and Mori and colleagues also suggested that this effect may be instinctual, protecting us from sources of danger such as corpses or unfamiliar animal species. Furthermore, this effect is amplified if the item has movement. Following on from Mori, the uncanny valley effect has been offered as an explanation for feelings of unease at other human-like artefacts such as dolls, masks, puppets, virtual reality avatars and computer-generated characters in movies (Seyama and Nagayama, 2007). Moore (2012) suggests a cognitive explanation for this phenomena; the emotional discomfort stems from the item causing ‘*sensory conflict*’ (p. 3) because it does not fit neatly into either of two item categories, e.g., the prosthetic hand is neither fully mechanical, nor fully human. With regard to clowns, the distortion of their facial features through makeup gives them a ‘near-human’ quality which may elicit the uncanny valley effect; however, no research has been conducted to explore this proposition. Relatedly, one suggestion in the popular psychology literature is that the fear of clowns stems from a difficulty in face processing and our subsequent inability to perceive the true emotions of the figure because of their makeup. This creates uncertainty as to the character’s intentions; the clown’s painted smile may be concealing anger and aggression and therefore be a potential threat to our safety (Flora, 2006; Christian, 2011).

Interestingly, the two key criteria suggested above—being a distorted version of a human figure or having concealed facial emotions—link to ideas that clowns are perceived as ‘creepy.’ Subjective creepiness has been defined as a feeling of anxiety triggered by uncertainty as to *‘whether there is something to fear or not and/or by the ambiguity of the precise nature of the threat* (e.g., *sexual, physical violence, contamination*)’ (McAndrew and Koehnke, 2016, p. 10). Indeed, in an exploration of the nature of creepiness using a survey of 1,341 participants, McAndrew and Koehnke (2016) reported that being a clown was rated as the creepiest occupation from a list of 21 possibilities, and this study indicated that a peculiar smile, bulging eyes, very pale skin and an odd dress sense were key features of creepiness. Facial characteristics, particularly eyes, eyebrows, teeth and lips were also associated with creepiness in a study by Watt et al. (2017), and the authors note how these areas are crucial for emotional transmission. Clown makeup which accentuates these features may therefore elicit a negative emotional response. In the personification of the “killer clown” as mentioned earlier, these facial characteristics are exaggerated further still, with these malevolent clowns bestowed with lines of sharp teeth and feral eyes. Such features may go beyond being merely unsettling or creepy and instead elicit a direct sense of threat.

The final appearance-related aspect of clown fear relates to disgust. This emotion has been defined as a ‘*revulsion response towards potential contamination*’ (Cisler et al., 2009, p. 36), from items or environments which are perceived to hold an infectious threat. A number of studies have identified revulsion as a feature of spider and blood injection injury phobias (Sawchuk et al., 2000; Cisler et al., 2009; Bianchi and Carter, 2012) and one study reported similar findings with specific phobias for wasps, bees and woodlice (Münstedt and Mühlhans, 2013). Importantly, it appears that disgust is a separate concept from fear, with the two emotions being distinguished physiologically, neurologically, cognitively and facially (Cisler et al., 2009). Disgust is also experienced more generally in response to items or situations that remind an individual of death (Bianchi and Carter, 2012); for example, people have a reluctance to sleep in a room in which someone had recently died, or to touch a dead body (Olatunji et al., 2007). In relation to fear of clowns, it is plausible that elements of clown makeup, with an emphasis on white face paint with bright red lips and nose, might be perceived as a contagious threat. Here, the white may act as a reminder of the pallor of the face of an unwell or deceased person (Stewart-Shaw, 2019), and the red; a reminder of the erythema that often co-occurs with infection.

Secondly, behavioural explanations of clown fear focus on the unpredictability of their behaviour, and like some of the explanations relating to physical characteristics, also relate to the concept of creepiness. McAndrew and Koehnke (2016) surveyed the mannerisms associated with creepiness, and here, unpredictability of behaviour was rated highly with participants responding affirmatively to the statement; ‘*I am uncomfortable because I cannot predict how he or she will behave*’ (McAndrew and Koehnke, 2016, p. 13). The wider literature on specific phobias also suggests that perceived unpredictability may be a key feature in eliciting alarm, and this has been demonstrated in studies with spider fearful individuals (Armfield and Mattiske, 1996; Vrijsen et al., 2009; Klahn et al., 2016). Furthermore, studies with children have reported fearful responses to toys which make unpredictable noises, such as laughing at random intervals (Gunnar et al., 1984; Yip et al., 2019). Clown behaviour might reasonably be considered unpredictable due to the nature of their performance where there is an emphasis on sleights of hand and magic tricks. Indeed, Durwin (2004) suggests that the fear of clowns is a cross-cultural phenomenon stemming from our unease at those who subvert social norms and break behavioural taboos. And again, when this erratic behaviour goes to extremes, it can be more than merely uncomfortable or unsettling and instead be perceived as directly threatening.

Thirdly, explanations for clown fear can be extrapolated from research into the development of other specific phobias, and relates to this phenomena stemming from learning and/or experience. Specifically, Rachman’s (1977) three pathways theory of fear acquisition suggest that the development of a specific phobia may be caused by; (1) direct experience, (2) observation (modelling) or (3) instruction/information. Here, the fear may be the result of an early frightening experience, may be ‘handed down’ from one generation to the next or might stem from negative media portrayals of clowns in popular culture (as described in the opening paragraphs). Rachman’s (1977) tripartite theory has some empirical support in the general specific phobia literature (King et al., 1998; Muris et al., 2008; Coelho and Purkis, 2009; Loxton et al., 2018).

In summary, within three broad categories, the preceding discussion has outlined eight plausible explanations for the origins of clown fear;

- Physical appearance
  1. *Their ‘proto’ human image makes us uneasy (the uncanny valley effect)*.
  2. *Their exaggerated facial features convey a direct sense of threat*.
  3. *Their makeup hides emotional signals so we cannot determine any harmful intent*.
  4. *Their makeup reminds us of death, infection or blood injury and thus evokes a disgust or avoidance response*.
- Behaviour
  5. *Their unpredictable behaviour makes us feel uncomfortable*.
- Learning and/or experience
  6. *The fear is modelled the fear from family members*.
  7. *The fear stems from negative portrayals of clowns in the media*.
  8. *The fear originated from a frightening experience with a clown*.

It is of course likely that a combination of the factors listed above may contribute to the fear of clowns. Indeed, several of the causative explanations may relate to an innate neurobiological fear response as is seen in other specific phobias (Garcia, 2017). Furthermore, there may be individual differences in the origins of such a fear. The methodology employed in this study will assist us in determining the relative contribution of each of the explanations, and enable us to look at associations between all the plausible causes. An original questionnaire was designed for this purpose, and this is the first study to focus specifically on the origins of coulrophobia.

### 1.3. The current study

This project had three key aims;

1. To assess the psychometric properties of a newly developed measure; the Origins of Fear of Clowns Questionnaire (OFCQ).
2. To explore the origins of clown fear in a sample of individuals who reported such a fear.
3. To explore the relationship between the demographic factors of gender and age on the self-reported origins of clown fear.

## 2. Methods

### 2.1. Participants

A snowball technique was used to recruit participants, with the survey being widely distributed both within the host institution, the University of South Wales, United Kingdom, and externally *via* social media. There was considerable emphasis on participants sharing the survey with international contacts in order for data to be captured from a wide population sample. After removing inadmissible responses, our survey sample consisted of 987 participants, of whom 790 (80%) were females and 197 (20%) were males. From this total population, it was primarily the participants who asserted that they had some degree of fear of clowns who were of interest in the current study, and this sample numbered 528; 53.5% of the total sample. Of this subgroup, 450 (85%) were female and 78 (15%) were male. The sample of participants affirming a fear of clowns included nationals of 52 different countries, although the majority were born in the United Kingdom. The age range for the utilised sample was between 18 and 72 (*M* = 28.20; SD = 9.95).

### 2.2. Materials

The survey consisted of an initial set of questions relating to key demographic information (i.e. gender, age). Participants were then asked if they were afraid of clowns with a dichotomous ‘Yes’ or ‘No’ response required. Additionally, the question ‘*How Afraid are you of clowns*’ was posed with options for responses being *‘Not at all,’ ‘Somewhat/Slightly,’ ‘Moderately’* or ‘*Extremely*.’ The data for the current study was drawn from the participants who answered that they were either *‘Somewhat/Slightly,’ ‘Moderately’ or ‘Extremely’* afraid of clowns (528 participants; 53.5%). Following this, an original questionnaire was presented for completion: the Origin of Fear of Clowns Questionnaire (OFCQ; see below). The questionnaire package was created and distributed using an online survey software platform.^1^

#### 2.2.1. Origin of Fear of Clowns Questionnaire

The Origin of Fear of Clowns Questionnaire (OFCQ) was designed to investigate the potential causative factors behind clown fear in participants who expressed such a fear. The research team collaborated to create the items to be included in the scale, based on the aetiological themes identified in the literature review above. The measurement tool was a 7-point Likert scale (1-Totally Disagree to 7-Totally Agree) incorporating 28 statements covering the 8 causative themes. In addition, 4 items were included which covered general physiological reactivity to clown stimuli giving a total of 32 items.

Examples of questionnaire items include;

*“I think clowns look disturbing”* (Physical Appearance: Uncanny Valley).

*“I cannot read a clown’s facial expression”* (Physical Appearance: Hidden Emotional Signals).

*“I worry a clown will do something unexpected”* (Behaviour: Unpredictability).

*“I have a significant family member or close friend who is afraid of clowns”* (Learning/Experience: Modelled from Family or Friend).

*“I have seen scary scenes in films involving clowns”* (Learning/Experience: Media Portrayals).

*“I feel my heart racing when I see a clown”* (General Physiological Reactivity).

This scale demonstrated high levels of reliability with an initial Cronbach’s alpha figure of 0.950. One item was deemed necessary to remove “*I was not afraid of clowns when I was younger*” due to an item-total correlation of −0.128 (the other item-total scores were all positive and ranged from 0.156 to 0.854). The Cronbach’s alpha figure increased to 0.955 with this item removed. The split-half reliability co-efficient for the amended 31-item OFCQ was 0.948.

### 2.3. Procedure

This study complied with the British Psychological Society Code of Human Research Ethics (British Psychological Society, 2014), and was granted ethical approval from the University of South Wales Faculty of Life Sciences and Education Ethics Committee. The project was advertised on several social media platforms, and *via* internal University messaging forums. After providing online consent, participants completed the demographic questions, fear and level of fear of questions and the Origin of Fear of Clowns Questionnaire (OFCQ). The survey was active between 16 September and 31 December 2019 and following cessation, data were exported to IBM SPSS Statistical Package for the Social Sciences (Version 26) for analysis.

### 2.4. Statistical analysis

Descriptive statistics consisting of frequencies and mean scores were used to present the demographic characteristics as a group and the gender split across each origin theme. Inferential analysis involved; (a) a Pearson’s r correlation involving age and origin themes, (b) a one-way Analysis of Variance with Bonferroni *post-hoc* comparisons to explore group differences between each origin theme, and (c) a Multivariate Analysis of Variance was used to explore the effect of gender across each origin theme. Finally, a factor analysis of the Origin of Fear of Clowns Questionnaire (OFCQ) was used to explore associations between the Likert items within and the factor structure of this measure.

## 3. Results

### 3.1. Comparison of origin categories

Considering the mean Likert scale scores for each of the eight causative themes, the ones which attracted the highest level of assent were; *Hidden Emotional Signals* (*M* = 5.20, *SD* = 1.44) and *Negative Media Portrayals* (*M* = 5.03, *SD* = 1.45), whilst items relating to *Modelling* (*M* = 2.78, *SD* = 1.62) and *Frightening Experience* (*M* = 2.65, *SD* = 2.03) attracted the lowest levels of agreement. Mean ratings and ranks for each causative theme are shown in Table 1.

**Table 1:** Mean ratings and ranking for causative themes with gender split.

Origin category	Causative theme	Mean score whole group (SD)	Rank order (whole group)	Mean gender split (Male vs. Female)
Physical appearance	*The Uncanny Valley Effect*	4.78 (1.55)	4	4.49 vs. 4.83
	*Direct Sense of Threat*	3.99 (1.75)	5	3.89 vs. 4.00
	*Hidden Emotional Signals*	5.20 (1.44)	1	4.91 vs. 5.25
	*Disgust/Avoidance*	3.40 (1.59)	6	3.25 vs. 3.43
Behaviour	*Unpredictable Behaviour*	4.96 (1.57)	3	4.55 vs. 5.03
Learning and /or Experience	*Modelling*	2.78 (1.62)	7	2.91 vs. 2.76
	*Negative Media Portrayals*	5.03 (1.45)	2	4.93 vs. 5.04
	*Frightening Experience*	2.65 (2.03)	8	2.73 vs. 2.63

Differences between these theme categories were statistically significant for the whole group according to a one-way ANOVA [*F*(7,4,216) = 214.847, *p* < 0.001], and Bonferroni *Post-Hoc* comparisons revealed that each mean score was statistically distinct from all others (*p* < 0.01 in all cases), except for *Uncanny Valley Effect* vs. Negative Media Portrayals (*p* = 0.359), and *Unpredictable Behaviour* (*p* = 1.00), *Negative Media Portrayals* vs. *Hidden Emotional Signals* (*p* = 1.00), and *Unpredictable Behaviour* (*p* = 1.00), *Modelling* vs. *Frightening Experience* (*p* = 1.00) and *Hidden Emotional Signals* vs. *Unpredictable Behaviour* (*p* = *0*.551). SPSS-adjusted *p*-values were reported here.

### 3.2. Demographic characteristics and origins of fear of clowns

As shown in Table 1, mean scores for all *Physical Appearance* and *Behaviour* items were marginally higher for females compared to males, whilst only one of the *Learning and/or Experience* items followed this pattern (*Negative Media Portrayals*). The other two items within this category (*Modelling, Frightening Experience*) showed the opposite pattern with males scoring higher than females. However, a Multivariate Analysis of Variance revealed no statistically significant effect of gender on origin themes *F*(8,519) = 1.93 *p* = 0.054; Wilks’ Lambda = 0.971, *η_p_^2^* = 0.03. There was likewise no significant difference in self-reported *Physiological Reactivity* between male and female participants, 3.39 vs. 3.66: *t*(525) = 1.09, *p* = 0.277.

Pearson’s *r* correlations were used to examine the association between age and the eight-origin theme mean scores on the OFCQ. When correcting for multiple tests by multiplying each reported *p* value by the number of correlations (9), only the association between age and Hidden Emotional Signals was statistically significant (*r* = 0.152, *N* = 521, *p* = 0.009). This indicates that as the age of participants increased, the mean score across this subscale also increased.

### 3.3. Factor analysis

A principal component analysis of the OFCQ using varimax rotation revealed a four-factor solution explaining 64.97% of the total variance. Eigenvalues of 14.50, 2.38, 2.06, and 1.20 were obtained for the four factors, accounting for 46.78, 7.67, 6.65, and 3.88% of the variance, respectively. The factor loading is shown in Table 2. Items are grouped by the original causative theme categories.

**Table 2:** Factor loadings and item-total correlations of the OFCQ. The bold factor loading coefficients indicate strong affiliation to each of the causative theme categories.

Item	Factor 1	Factor 2	Factor 3	Factor 4	TOTAL IC
Uncanny valley effect					
I think clowns look disturbing	**0.620**	0.355	0.359		0.705
Clowns look odd and often seem very out of place	**0.552**	0.428	0.383		0.709
Clowns make me feel uneasy	**0.781**	0.365			0.824
I think clowns seem more like demons or aliens than humans	**0.585**	0.321			0.673
Direct sense of threat					
I cannot remember a time when I was not afraid of clowns	**0.669**				0.576
If I saw a clown, I would be on edge	**0.818**	0.321			0.854
I worry that a clown might harm me	**0.685**	0.361			0.789
Clowns seem like a potential threat	**0.691**	0.421			0.820
If I saw a clown, I could not help focusing my attention on it	**0.544**	0.401			0.683
Hidden emotional signals					
I cannot tell what a clown is thinking		**0.739**			0.534
I find it difficult to read a clown’s facial expression		**0.739**			0.584
When someone is dressed as a clown, I cannot tell who is underneath		**0.712**			0.527
Disgust/avoidance					
I would not want a clown to be in close proximity to me	**0.740**	0.368			0.812
Clowns make me feel sick to my stomach	**0.834**				0.729
I cannot stand the sight of clowns	**0.857**				0.811
Clowns remind me of disease and illness	0.347			**0.680**	0.401
Unpredictable behaviour					
If I saw a clown, I could not tell how the clown will behave	0.438	**0.663**			0.692
I worry a clown will do something unexpected	**0.619**	0.506			0.755
I cannot tell if a clown is friendly or if they will make fun of me	0.455	**0.634**			0.675
I think clowns are creepy	**0.573**	0.416	0.381		0.705
Modelling					
A parent or family member has told me to be wary of clowns				**0.786**	0.302
I have a significant family member or close friend who is afraid of clowns			0.357	**0.508**	0.156
Negative media portrayals					
I have read stories in the news about people dressed as clowns harming others			**0.743**		0.406
I have seen videos within the media about killer clowns			**0.766**		0.392
I have read books involving killer clowns			0.438	**0.502**	0.198
I have seen scary scenes in films involving clowns			**0.632**		0.276
Frightening experience					
I have had a negative experience with a clown	**0.516**			0.464	0.472
I was not afraid of clowns when I was younger*					−0.128
Physiological reactivity					
If I saw a clown, this would make my muscles tense up	**0.867**				0.844
Clowns make me break out in a sweat	**0.869**				0.787
I feel my heart racing when I see a clown	**0.883**				0.828
Clowns make my skin crawl	**0.825**				0.803

*This item shown here for completeness, but was omitted prior to PCA.

Factors 2 and 3 readily lend themselves to conceptual categories and correspond fairly well to our putative causal themes: Factor 2 items clearly relate to *“unpredictability of behaviour and uncertainty about harmful intent”* (unpredictable behaviour and hidden emotional signals). Factor 3 items related solely to “*negative media portrayals*.” Of the items loading most heavily onto Factor 4, two items relate to direct instruction or modelling from family, a third relates to influence from reading books, so this factor could be identified as “*familial or cultural transmission*.” However, the fourth item that loaded most heavily onto this factor; ‘Clowns remind me of disease and illness,’ complicates the picture somewhat. As the majority of the items loaded onto Factor 1, it is somewhat more difficult to conceptually characterise this factor, since the items originated from different putative causal themes based on our review of the literature. However, the majority of these items relate to physiological reactivity (e.g., I feel my heart racing when I see a clown), disgust (e.g., clowns make me feel sick to my stomach), direct sense of threat (e.g., I worry that a clown might harm me) and the uncanny valley effect (e.g., I think clowns look disturbing). We could say therefore that this factor may represent “*visceral reactions to the grotesque or unusual*.”

## 4. Discussion

This study set out to investigate the origins of coulrophobia, and for this purpose, we constructed the Origins of Fear of Clowns Questionnaire (OFCQ) to measure the extent to which a range of hypothetical causal candidates contribute to clown fear. Results from this instrument found broad support for all theorised aetiological factors to varying degrees. *Hidden Emotional Signals, Negative Media Portrayals, Unpredictable Behaviour* and the *Uncanny Valley Effect* attracted the highest ratings of agreement in respective rank order. These were followed by *Direct Sense of Threat*, *Disgust/Avoidance* and *Modelling*. Perhaps surprisingly, the lowest level of agreement was for questions relating to having had a *Frightening Experience* in the presence of a clown; indicating that simple, direct conditioning alone is an insufficient explanation of clown fear in the majority of individuals. However, what is unclear at this stage is the level of fear associated with each origin category and this is the focus of continued research.

**Figure 1:**
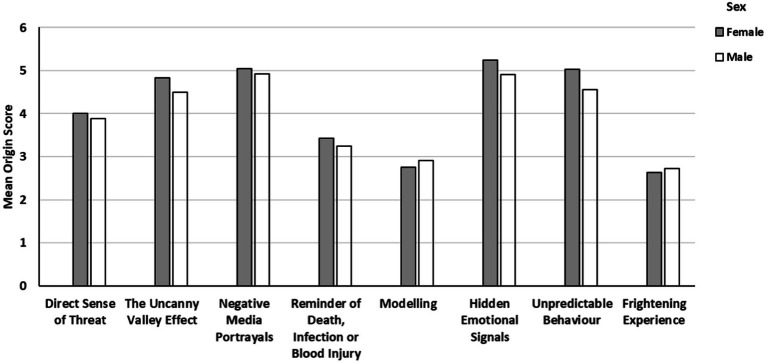
Mean origin subscale scores between males and females who self-reported somewhat/slightly afraid of clowns or above. None of the gender comparisons were statistically significant, but the consistency of responses to each causative theme is clearly illustrated.

No statistically significant sex differences were observed for any of the origin categories, suggesting that common aetiological factors account for coulrophobia in both males and females, and this is illustrated in Figure 1. Again this finding is somewhat surprising given that sex differences in fear are widely reported (e.g., Cornelius and Averill, 1983; Ramikie and Ressler, 2018; Day and Stevenson, 2020). Relatedly, females typically show greater disgust responses than males (Al-Shawaf et al., 2018), so a more prominent difference on the *Disgust/Avoidance* subscale in particular was anticipated. The sex imbalance (85% F, 15% M) in our sample could account for this lack of difference to an extent. There were no statistically significant correlations between age and causative themes except for *Hidden Emotional Signals*, indicating that as age increases, so does uncertainty about a clown’s harmful intent. This could indicate a greater vigilance for potential threat or greater difficulty in emotional processing with advancing age.

Our factor analysis of the OFCQ supported a multifactorial explanation, and confirmed the importance of media influences (i.e. a learned/instructed mechanism) and the unpredictability/uncertainty about harmful intent (i.e. a cognitive mechanism) as important aetiological contributors. Such explanations suggest a rational basis for the fear of clowns; if for instance one has reason to suspect harm in the presence of a specific stimulus (having observed or been instructed about erratic or overtly threatening behaviour) then an aversion to that stimulus is rational. Rachman (1977) suggested that fears are acquired through one or a combination of pathways: (1) direct conditioning, (2) vicarious learning, and (3) negative information/instruction. Our findings provide some support for all three pathways in the development of clown fear; less so for the direct pathway, but more so for information-induced fear, e.g., through the media and popular culture. Fright-induced media content can have sustaining effects into adulthood consequently from exposure as a child or teenager (Custers and Van den Bulck, 2011) and many retrospective studies examining undergraduates’ memories of emotional reactions to media support this view (Harrison and Cantor, 1999; Hoekstra et al., 1999; Cantor, 2004).

However, other stimuli evoke fear reactions without prior training (unconditioned fear stimuli), and indeed, most items from the OFCQ loaded onto a factor which represented a more emotional and autonomic reaction to clowns, encompassing the items relating to heightened physiological reactivity, a more ambiguous sense of threat and feelings of unease or revulsion. This suggests that there is also a more instinctive “gut” response involved in clown fear, akin to that experienced in the presence of fear-relevant stimuli such as snakes or spiders. Non-associative theoretical accounts argue that direct or indirect conditioning events are not required for the onset of fear responses to stimuli that have some type of evolutionary or biological significance (Menzies and Clarke, 1995). Thus, the aversive pairing of an unconditioned stimulus to an unconditioned response is no longer the *sine qua non* for fear acquisition (Mackintosh, 1983).

The question that then follows is, *why* should a clown elicit such instinctual reactions? What are the stimulus properties of clowns that engender such a response? A clown is a compound stimulus consisting of many different individual elements. It may not be any of these individual elements that is in itself frightening, but rather the juxtaposition of these features. That said, some of the clowns that anecdotally are often described as scary (including the aforementioned Pennywise, who is meant to be so, but also including Ronald McDonald, who is not) do share some common physical attributes—a full face of white makeup with red accents, with no flesh tones visible underneath, and red hair. Indeed as mentioned earlier, the literature has already proffered a number of prominent theories of clown fear consistent with such observations. Firstly, the uncanny valley effect—because clowns are not-quite-human in appearance, and this effect may be amplified by makeup that completely covers the skin or exaggerates certain facial features—and secondly, the particular combination of pallor and redness in a clown’s makeup is reminiscent of disease and contagion. These factors can combine to give a clown an appearance of deformity, to which (sadly, but nevertheless unavoidably) humans have a natural reaction of revulsion and fear. Prior research has found that faces with mismatched or disproportionate facial features (e.g., bulbous nose, protruding forehead) can produce unease/unpleasant feelings in participants (Seyama and Nagayama, 2007) and MacDorman et al. (2009) suggest such feelings are deeply rooted in the basic emotion of disgust because of the human mechanism of pathogen avoidance. Our results now provide some corroboratory evidence to support these prior hypotheses in the literature. Future research could provide a stronger test of these hypotheses by, for instance, presenting participants with a series of images of clowns, varying the colour and configuration of their face makeup and hair colour, and whether the makeup fully or partially covers the face and then measuring subsequent reactions to these stimuli. Finally, one important area that was not considered within the current study is the link between adverse childhood experiences and coulrophobia. There is evidence to suggest that early trauma is linked to anxiety disorders in adulthood, including social phobia (e.g., Bruijnen et al., 2019), and some negative clown characteristics, such as unpredictability or sense of threat, may be features of an early abusive relationship. Therefore in individuals who have suffered trauma and adversity, a clown may trigger their fears from childhood. This should be another focus for future research.

This study is not without limitations. Further investigation using equal numbers of males and females is warranted. Indeed, only 15% of our sample were males and an important consideration for any study in this area is the evidence of sex differences in the fear response (Lebron-Milad et al., 2012). Therefore studies should be cautious in drawing general conclusions from a mixed sample, especially one with such a gender imbalance. Nevertheless, there was a remarkable degree of consistency between male and female participants in the mean origin scores for each causative theme as can be seen in Figure 1. A second limitation relates to the severity of clown fear and how this might be related to aetiological factors. Indeed, we do not know if any of our participants would meet the diagnostic criteria for a specific phobia according to the DSM-5 (American Psychiatric Association, 2013) and therefore we are unable to determine the extent to which a clinically recognised fear is associated with specific aetiological factors. Relatedly, it would have been also useful to consider associations between fear of clowns and other fears/phobias, and indeed other mental health problems such as anxiety and depression. Future research should consider comorbidity and common aetiological factors associated with all co-occurring mental health conditions. Another focus for further study should be in obtaining autobiographical narrative accounts of the origins of clown fear through interviews. This methodology would enable the collection of rich, qualitative data and provide further important insight into this intriguing, and little understood, phenomena.

In conclusion, this study is the first to investigate the aetiology of clown fear and to consider competing explanations of the origins of this phenomena. We constructed an original questionnaire for this purpose, and our findings suggest that media influences, unpredictability of behaviour and uncertainty about harmful intent play an important role in the origins of coulrophobia. There are also multiple features of clown appearance which produce a negative experiential state and a sense of a direct threat. Certainly our findings offer an explanation as to why clowns feature as figures of fear within popular culture.

## Data availability statement

The raw data supporting the conclusions of this article will be made available by the authors, without undue reservation.

## Ethics statement

The studies involving human participants were reviewed and approved by the University of South Wales, Faculty of Life Sciences and Education Ethics Committee. The patients/participants provided their written informed consent to participate in this study.

## Author contributions

PT, SD, and WG: writing – original draft and writing – review and editing. SS: original data collection, writing – original draft, and writing – review and editing. All authors approved the final version of the article.

## Conflict of interest

The authors declare that the research was conducted in the absence of any commercial or financial relationships that could be construed as a potential conflict of interest.

## Publisher’s note

All claims expressed in this article are solely those of the authors and do not necessarily represent those of their affiliated organizations, or those of the publisher, the editors and the reviewers. Any product that may be evaluated in this article, or claim that may be made by its manufacturer, is not guaranteed or endorsed by the publisher.

## Footnotes

^1^ https://www.onlinesurveys.ac.uk/
